# Predictors for Early Identification of Hepatitis C Virus Infection

**DOI:** 10.1155/2015/429290

**Published:** 2015-08-27

**Authors:** Mei-Hua Tsai, Kuei-Hsiang Lin, Kuan-Tsou Lin, Chi-Ming Hung, Hung-Shiang Cheng, Yu-Chang Tyan, Hui-Wen Huang, Bintou Sanno-Duanda, Ming-Hui Yang, Shyng-Shiou Yuan, Pei-Yu Chu

**Affiliations:** ^1^Kaohsiung Blood Center, Taiwan Blood Services Foundation, Kaohsiung 811, Taiwan; ^2^Department of Medical Laboratory Science and Biotechnology, College of Health Sciences, Kaohsiung Medical University, Kaohsiung 807, Taiwan; ^3^Department of Clinical Laboratory, School of Medicine, College of Medicine, Kaohsiung Medical University, Kaohsiung 807, Taiwan; ^4^Cishan Hospital, Ministry of Health and Welfare, Kaohsiung 842, Taiwan; ^5^Department of Medical Imaging and Radiological Sciences, College of Health Sciences, Kaohsiung Medical University, Kaohsiung 807, Taiwan; ^6^Translational Research Center, Kaohsiung Medical University Hospital, Kaohsiung 807, Taiwan; ^7^Institute of Medical Science and Technology, National Sun Yat-Sen University, Kaohsiung 804, Taiwan; ^8^Department of Anesthesiology, Chang Gung Memorial Hospital-Kaohsiung Medical Center, Chang Gung University College of Medicine, Kaohsiung 833, Taiwan; ^9^Department of Biological Sciences, National Sun Yat-Sen University, Kaohsiung 804, Taiwan; ^10^Edward Francis Small Teaching Hospital, Banjul 1515, Gambia; ^11^Department of Medical Research, Kaohsiung Medical University Hospital, Kaohsiung 807, Taiwan; ^12^Department of Obstetrics and Gynecology, Kaohsiung Medical University Hospital, Kaohsiung 807, Taiwan; ^13^School of Medicine, College of Medicine, Kaohsiung Medical University, Kaohsiung 807, Taiwan; ^14^Department of Laboratory Medicine, Kaohsiung Medical University Hospital, Kaohsiung 807, Taiwan

## Abstract

Hepatitis C virus (HCV) infection can cause permanent liver damage and hepatocellular carcinoma, and deaths related to HCV deaths have recently increased. Chronic HCV infection is often undiagnosed such that the virus remains infective and transmissible. Identifying HCV infection early is essential for limiting its spread, but distinguishing individuals who require further HCV tests is very challenging. Besides identifying high-risk populations, an optimal subset of indices for routine examination is needed to identify HCV screening candidates. Therefore, this study analyzed data from 312 randomly chosen blood donors, including 144 anti-HCV-positive donors and 168 anti-HCV-negative donors. The HCV viral load in each sample was measured by real-time polymerase chain reaction method. Receiver operating characteristic curves were used to find the optimal cell blood counts and thrombopoietin measurements for screening purposes. Correlations with values for key indices and viral load were also determined. Strong predictors of HCV infection were found by using receiver operating characteristics curves to analyze the optimal subsets among red blood cells, monocytes, platelet counts, platelet large cell ratios, and mean corpuscular hemoglobin concentrations. Sensitivity, specificity, and area under the receiver operator characteristic curve (*P* < 0.0001) were 75.6%, 78.5%, and 0.859, respectively.

## 1. Introduction

According to the World Health Organization, deaths from primary hepatocellular carcinoma (HCC) exceeded 1 million in 2010. The leading risk factors for HCC are hepatitis B virus (HBV) and hepatitis C virus (HCV) infections [1]. HCC is known to be the most common form of liver cancer and the third leading cause of cancer-related deaths worldwide [2]. Unlike HBV infection, at present, there is no vaccine to prevent HCV infection approved by the Food and Drug Administration (FDA) [3, 4]. The global prevalence of HCV antibodies is 0.5%–2% and, in Taiwan, the average seropositive rate is 4.4%, but may exceed 30% in mountainous or coastal regions [5–7]. Further, genotype 1b is reportedly a major risk factor for HCC [8]. In southern Taiwan, the reported prevalence of HCV genotype 1b is 50–60% and that of 2a is 30–40% [8]. These figures are consistent with an earlier international survey of HCV in blood donors, which revealed that 57% were infected with HCV type 1 and 43% were infected with HCV type b [9]. The increased morbidity and mortality rate prevailing in HCV infection and the increased burden of HCV-related infections have attracted widespread attention, with potential slow evolving characteristics at rapidly progressive signatures causing greater impact on public health [10–13]. Risk factors include frequent exposure to blood or contaminated needles. In the USA, birth during the baby boom era (1945–1965) was also recommended for inclusion as an HCC risk factor [14]. One reason why HCV remains a major public health threat is the difficulty of developing an effective treatment strategy because HCV is characterized by long episodes during which the patient is asymptomatic, even after liver damage has occurred.

In a high percentage (54%–86%) of cases, HCV infection persists for many decades and ultimately causes liver cirrhosis or HCC [15]. Therefore, early detection of HCV infection is essential not only to enable initiation of HCV therapy sufficiently early to prevent disease progression but also to prevent further transmission. Recombinant immunoblot assay (RIBA) and nucleic acid test (NAT) methods have relatively high specificity for detecting HCV but are not cost effective [12, 14, 16]. Although developing a test for identifying HCV at an early stage is challenging, such a test is urgently needed for initially identifying HCV in endemic geographic locations where the risk is high. In haematopoietic cells, HCV interferes with peripheral blood maturation and causes thrombocytopenia [17]. Thrombocytopenia also occurs in HCV infection and in liver cirrhosis [18–21]. Notably, patients with liver fibrosis or cirrhosis have abnormally low serum thrombopoietin (TPO) levels [22] since TPO is mostly produced by the liver before its release into the bloodstream [23]. Thrombopoietin is the main regulator of platelet production, and a feedback loop between circulating TPO and platelet mass has been reported [24]. However, little is known regarding platelet count (PLT) and TPO levels in apparently healthy people.

A complete blood count (CBC) is one of the most commonly performed blood tests. Since it reveals peripheral blood changes, the CBC is routinely performed in health examinations, even in asymptomatic patients. However, there is no evaluation showing the screen for HCV potential infection by CBC data. The objective of this study was to identify an optimal subset of routinely obtained haematological indices that can be used to discriminate potential HCV infection cases from the general population. Further, the change of TPO levels in apparently healthy people was also examined.

## 2. Materials and Methods

### 2.1. Subjects

Blood samples were obtained from the Kaohsiung Blood Center between January 2008 and December 2009. Before transfusion, all blood donors were required to complete a “Blood Donor Registration Form” http://intra.blood.org.tw/upload/b1f25843-f5f6-4c91-b483-6b81d417a133.pdf and to undergo an interview regarding health condition and lifestyle in order to estimate blood quality. The blood samples were subjected to tests for transfusion-transmissible infections, including the RPR and TPPA tests for syphilis and tests for HBsAg, anti-HCV, and anti-HTLV I/II. In each of the 144 randomly selected subjects in the experimental group in this study, HBsAg (−), anti-HIV (−), anti-HTLV I/II (−), anti-HCV (+), or HCV which was confirmed by sera positive for anti-HCV and positive for either HCV RNA or RIBA. The other 168 randomly selected subjects with HBsAg (−), anti-HIV (−), anti-HCV (−), or anti-HTLV I/II (−) infection were enrolled in the negative control group. No data were collected until signed consent forms were received from each participant. This study was approved by the Ethics Committee of the Taiwan Blood Services Foundation.

### 2.2. Laboratory Tests

Anti-HCV-positive cases were identified by a Murex anti-HCV (Version 4.0) enzyme immunoassay (Abbott, South Africa) in enzyme-linked immunosorbent assay (ELISA). The following measurements were performed according to the manufacturer instructions: CBC counts were measured with a Sysmex XT-1800i autoanalyser (Sysmex, Japan), alanine aminotransferase (ALT) levels were measured with an AU7200 autoanalyser (Beckman Coulter, USA), and serum TPO levels were measured by ELISA (Quantikine; R&D Systems Europe, Oxfordshire, UK). The HCV RNA viral loads were measured by real-time PCR using the COBAS AmpliPrep/COBAS TaqMan HCV Test (Roche Molecular Systems, USA). A RIBA (Chiron RIBA HCV 3.0 Strip Immunoblot Assay, Novartis Vaccines and Diagnostics, USA) was performed to verify positive responses to anti-HCV.

### 2.3. Statistical Analysis

Statistical analyses were performed using JMP software (Version 9.0, SAS Institute Inc., Cary, NC, USA). Chi-square test and Student's *t*-test were used to compare demographic characteristics and clinical measurements between the HCV-positive experimental group and the HCV-negative control group. The analysis of covariance (ANCOVA) was used to compare haematological indices, including alanine transaminase (ALT) and TPO levels, between the 2 groups, with adjustment for gender and age. Stepwise multiple logistic regression analysis was used to evaluate variables for associations with HCV infection. Receiver operating characteristic (ROC) analysis was performed to calculate the sensitivity and specificity, area under the ROC curve (AUROC), and the optimal cut-off value. Correlations between key parameters and HCV viral loads were calculated using Spearman correlation. A *P* value less than 0.05 was considered statically significant.

## 3. Results

### 3.1. Study Population

Table 1 shows the basic demographic characteristics of participants in the HCV-infected group (83 men and 61 women; mean age = 39.3 ± 10.8 y) and in the negative control group (38 men and 130 women; mean age = 37.4 ± 7.3 y). Out of 144 HCV-positive cases, 24 (16.7%) showed serum HCV RNA negativity. The median (interquartile range, IQR) HCV viral load was 517556.0 (10119.5–3418142.0) IU/mL.

### 3.2. Effects of HCV Infection on Haematological Indices

Table 1 also shows that platelet-related indices significantly differed between the HCV-infected group and the negative control group. Compared with the control group, the HCV-infected group had significantly lower platelet counts (PLT) and plateletcrit counts (PCT) but significantly higher platelet distribution widths (PDW), mean platelet volumes (MPV), and platelet-large cell ratios (P-LCR). Restated, the HCV-infected group had lower PLT and PCT but higher MPV, PDW, and P-LCR. This implied that the HCV-infected group had larger, more irregular, and more numerous platelets compared to the controls.

Compared with the negative control group, the HCV-infected group showed significantly higher red blood cell counts (RBC), haemoglobin (Hb) levels, and haematocrit (HCT) levels (Table 1). Compared with the control group, the HCV-infected group also showed significantly higher white blood cell counts (WBC), lymphocyte counts, and monocyte counts (MONO). Notably, Hb, HCT, and all four cell counts (RBC, WBC, lymphocytes, and MONO) were higher in the HCV-infected group than in the control group.

### 3.3. Effects of HCV Infection on TPO and ALT Levels

Mean TPO levels were significantly higher in the HCV-infected group than in the negative control group (74.0 ± 66.55 pg/mL versus 42.04 ± 37.89 pg/mL, resp.; *t*-test, *P* < 0.001; ANCOVA, *P* < 0.001; Mann-Whitney, *P* < 0.0001). However, mean ALT levels did not significantly differ between the HCV-infected group and the negative control group (35.5 ± 45.0 IU/L versus 23.3 ± 29.5 IU/L, resp.; *t*-test, *P* = 0.006; ANCOVA, *P* = 0.339; Mann-Whitney, *P* < 0.0001).

### 3.4. Comparative Prediction Performance of Haematological Indices

Stepwise multiple logistic regression analysis was performed to identify haematological indices, ALT factors, and TPO factors that predict HCV infection. The HCV infection status was used as the dependent variable; haematological indices, ALT, and TPO were used as independent variables. The model revealed seven significant predictors: mean corpuscular haemoglobin concentration (MCHC), RBC, PLT, MPV, P-LCR, MONO, and TPO (Table 2). Analysis of the comparative effects of values for these key factors and HCV viral load showed that PLT had a significant negative correlation with viral load (*r* = −0.333, *P* < 0.001) (Table 3). The HCV viral load had significant positive correlations with TPO and MONO (*r* = 0.351, *P* < 0.001 and *r* = 0.370, *P* < 0.001, resp.). That is, patients with elevated HCV viral loads had decreased PLT whereas those with decreased PLT had elevated TPO.

An ROC analysis was then performed to determine the optimal cut-offs for the factors in a dummy-variable logistic regression model. Binary variables were coded as 1 or 0. A stepwise procedure was used to select an optimal subset of dummy regressors and point scores. Finally, six dummy variables that showed significant differences (RBC, MCHC, PLT, P-LCR, MONO, and TPO) were identified as predictors of HCV infection and were used to construct the AUROC (range = 0.651–0.741) (Table 4).

**Figure 1:**
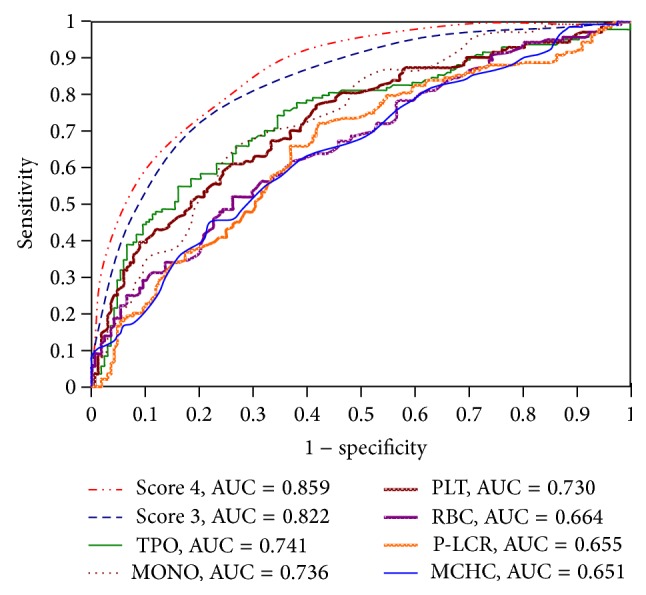
The ROC curve analysis of scores with best prognostic power for predicting HCV infection.

This method accounted for the point score whereas the final score (range = 1–7) was the sum of the parameters. A score of 4, which was considered optimal, yielded a sensitivity of 75.6% and a specificity of 78.5%. The AUROC for HCV infection was 0.859 (*P* < 0.0001) (Figure 1). Excluding TPO, since this indicator is not performed in routine, the haematological indices routinely used to predict HCV infection (RBC, MCHC, PLT, P-LCR, and MONO) had values ranging from 1 to 5; a score of 3, which was considered optimal, yielded a sensitivity of 76.8% and a specificity of 75.7%. The AUROC for HCV infection was 0.822 (*P* < 0.0001) (Figure 1).

## 4. Discussion

Since previous studies indicate that thrombocytopenia results from chronic liver disease, we speculated that a haematological comparison between a healthy blood donor and a donor with HCV might reveal the impact of HCV on PLT and TPO; an improved understanding of this impact could help determine whether a donor has HCV. This hypothesis was tested by investigating the relationships among haematological indices and TPO and HCV viral loads. The haematological indices and TPO were also evaluated in terms of predictive performance. Because of the varying consent given by the participants, the negative control group and HCV-infected group were not matched by age or gender. Therefore, ANCOVA was used to adjust the statistical analysis for age and gender.

The data analysis indicated that ALT levels were not significantly associated with HCV infection. The ALT levels were abnormal in most hepatitis patients [25] and in most (70%–80%) HCV carriers [26]. Identifying HCV is often difficult because HCV carriers with persistently normal ALT levels are usually asymptomatic or have nonspecific symptoms. Meanwhile, MPV is not a reliable indicator as its value is known to change over time in the presence of ethylenediaminetetraacetic acid (EDTA) anticoagulant. Studies indicate that 15% to 25% of the people who recover from an initial HCV infection have persistent antibodies in the absence of the virus [27]. Thus, the 16.7% of cases who revealed positive RIBA and negative RNA in the serum HCV test in this study were most likely cases that had recovered. Further, since the window between HCV infection and detectability can exceed 70 days [28], some participants classified as anti-HCV-negative in this study may have actually been infected with HCV. Therefore, improved tools for monitoring HCV are urgently needed to minimize bias.

In a previous study, an HCV-infected group, which also had chronic hepatitis and cirrhosis, had a mean PLT greater than 150 × 10^3^ *μ*L [20, 29]. In contrast, the mean PLT in the HCV-infected group in this study was significantly lower than that in the control group. This difference likely resulted from the relatively lower severity of infection in the HCV-infected group in the present study, since the average seropositive rate in blood donors is much lower than that in the general population in Taiwan (0.07% versus 4.4%, resp.) [30, 31]. Thrombocytopenia is commonly defined as a PLT below 50,000 per microlitre, which is a level commonly observed in cases of viral infection. Hypothesized mechanisms of thrombocytopenia include (1) dissemination by intravascular coagulopathies [32], (2) impairment of thrombocytopoiesis by a megakaryocyte mutation caused by the virus [21, 33, 34], (3) direct interaction between the virus and platelets in blood circulation (e.g., phagocytosis or the aggregation, release, and thrombocytosis of platelets) [35, 36], (4) an antigen-antibody complex that impairs platelet or antiplatelet antibodies that directly antagonise platelet-specific antibodies (e.g., anti-i) [37–39], and (5) a virally caused depletion of sialic acid (containing neuraminic acids) on platelet surfaces [40]. Thrombocytopenia has also been reported in viral infections such as herpes simplex virus, vaccinia virus, human immunodeficiency virus, hantavirus, dengue II virus, and influenza virus [35, 37, 41–44]. In the current study, MPV, PDW, and P-LCR were higher in the HCV-infected group than in the control group, which indicates that large platelet formation is an inappropriate bone marrow response to thrombocytopenia.

Unlike liver cirrhosis, the mechanism of decreased PLT in HCV is decreased TPO secretion [22, 23]. In this study, platelet counts were negatively correlated with TPO levels in the HCV-infected group. Since TPO regulates the maturation of megakaryocytes and thrombocytopoiesis, increased TPO secretion which is a normal physiological response to reduced PLT might elevate PLT in blood circulation. Therefore, the reduced PLT observed in the blood circulation of the HCV infected group in this study might have resulted from increased TPO levels. In cases with both positive HCV antibodies and low PLT, HCV viremia can reportedly be predicted with 88.8% accuracy [45]. The HCV RNA viral load is a useful indicator for monitoring the course of HCV and treatment efficacy [46]. In this study, HCV viral loads were negatively correlated with PLT but positively correlated with TPO. Previous studies of HCV infection revealed that the virus binds to glycoproteins on platelet surfaces; glycoprotein apparently plays a role in viral transport and in the persistence of ligands on platelet surfaces [39, 47]. Therefore, we speculate that HCV affects the PLT in circulating blood, which then affects TPO secretion.

The ROC curve analysis is widely used to measure the discriminatory power of diagnostic or prognostic tests [48]. In the current study, ROC curve analysis revealed that the optimal subset of haematological indices (RBC, MCHC, PLT, P-LCR, and MONO) had an AUC of 0.822 (specificity = 0.768; sensitivity = 0.757), which confirmed that this subset was a significant predictor of HCV. Some patients classified as having suspected HCV infection in this study had normal to low-normal RBC and PLT values. Again, however, some cases included in this analysis may have been classified as “healthy” because they had not yet been excluded by a blood donation screening test. One solution is the use of a computer program to improve accuracy in screening candidates for further HCV confirmatory tests.

## 5. Conclusions

Hepatitis C virus is the main risk factor for HCC in Western Europe, North America, and Asia. Almost all HCV-associated HCCs occur in patients with cirrhosis. Antiviral treatment is the only available option for preventing or delaying the occurrence of HCC in patients with chronic HCV infection. In the early stages of HCC, malignant behavior may not have a strong correlation with histological appearance. The use of improved HCV screening methods that can detect infection in early stage not only limits further spread but also reduces the overall number of chronic HCV cases and substantially reduces the incidence of HCC.

Currently, the most urgent tasks are identifying potential markers for screening or early diagnosis of HCC among high-risk individuals with chronic hepatitis C and identifying target molecules for the treatment and prevention of HCV-associated HCC. The analytical results of this study suggest that cases in which RBC, MCHC, PLT, P-LCR, and MONO exceed the optimal cut-off values require further confirmation such as by HCV NAT. The data obtained in this study can be used to improve accuracy in screening the general population for potential cases of HCV infection. To enhance the early detection of HCV infection, further studies are needed to modify and improve existing screening procedures and to develop convenient supplemental screening flowcharts. Until then, the findings of this study should be applied cautiously.

## Figures and Tables

**Table 1:** Comparison of demographic characteristics and clinical measurements in the HCV-infected group and in the negative control group.

Variable	HCV-infected group (*n* = 144)	Negative control group (*n* = 168)	*t-*test *P*	ANCOVA *P* ^#^
Gender *n* (%)				
Male	83 (57.6)	38 (22.6)		
Female	61 (42.4)	130 (77.4)	<0.001	
Age mean (sd)	39.3 ± 10.8	37.4 ± 7.3	0.071	
WBC (×10^3^ *μ*l)	6.8 ± 1.9	6.0 ± 1.7	<0.001	0.006
RBC (×10^6^ *μ*l)	4.9 ± 0.7	4.5 ± 0.5	<0.001	0.024
HB (g/dl)	14.7 ± 1.5	13.4 ± 1.8	<0.001	<0.001
HCT (%)	43.0 ± 3.9	39.8 ± 4.4	<0.001	0.001
MCV (fl)	89.1 ± 8.0	88.1 ± 6.3	0.243	0.122
MCH (pg)	30.5 ± 3.1	29.6 ± 2.8	0.008	0.030
MCHC (g/dl)	34.2 ± 1.0	33.5 ± 1.3	<0.001	0.005
PLT (×10^3^ *μ*l)	222.3 ± 58.8	270.5 ± 61.8	<0.001	<0.001
NEU (%)	56.4 ± 9.0	58.3 ± 8.2	0.052	<0.001
LYM (%)	34.0 ± 8.2	33.1 ± 7.7	0.270	0.648
MONO (%)	6.6 ± 1.8	5.7 ± 1.4	<0.001	<0.001
EOS (%)	2.5 ± 1.6	2.4 ± 1.7	0.630	0.174
BAS (%)	0.5 ± 0.3	0.5 ± 0.3	0.140	0.230
RDW-SD (fl)	41.8 ± 2.9	42.6 ± 2.9	0.013	0.055
RDW-CV (%)	13.3 ± 1.5	13.6 ± 1.5	0.040	0.034
PDW (fl)	13.2 ± 2.0	12.1 ± 1.9	<0.001	<0.001
MPV (fl)	10.8 ± 0.8	10.5 ± 0.9	<0.001	0.001
P-LCR (%)	31.7 ± 6.2	28.5 ± 6.4	<0.001	<0.001
PCT (%)	0.2 ± 0.1	0.3 ± 0.1	<0.001	<0.001
NEUT (×10^3^ *μ*l)	3.9 ± 1.4	3.6 ± 1.3	0.034	0.090
LYMPH (×10^3^ *μ*l)	2.3 ± 0.7	1.9 ± 0.6	<0.001	0.004
MONO (×10^3^ *μ*l)	0.4 ± 0.1	0.3 ± 0.1	<0.001	<0.001
EOS (×10^3^ *μ*l)	0.2 ± 0.1	0.1 ± 0.1	0.083	0.561
BAS (×10^3^ *μ*l)	0.03 ± 0.02	0.03 ± 0.02	0.463	0.789
TPO (pg/ml)	74.4 ± 66.3	42.0 ± 37.9	<0.001	<0.001
ALT (IU/L)	35.5 ± 45.0	23.3 ± 29.5	0.006	0.339

HCV: hepatitis C virus; ANCOVA: analysis of covariance; WBC: white blood cell count; RBC: red blood cell count; Hb: haemoglobin; HCT: haematocrit; MCV: mean corpuscular volume; MCH: mean corpuscular haemoglobin; MCHC: mean corpuscular haemoglobin concentration; RDW: RBC distribution width; PLT: platelet count; PCT: plateletcrit; PDW: platelet distribution width; MPV: mean platelet volume; P-LCR: platelet-large cell ratio; NEU: neutrophil; LYM: lymphocyte; MON: monocyte; EOS: eosinophil; BAS: basophil; TPO: thrombopoietin; ALT: alanine aminotransferase.

Significance level: *P* < 0.05.

^#^Age- and gender-adjusted *P*value.

**Table 2:** Results of multivariate stepwise regression analysis.

Variables	Odds Ratio	95% CI	*P*
RBC ≥ 4.76 (×10^6^ *μ*l)	2.043	1.104–3.810	0.023
MCHC ≥ 33.9 (g/dl)	2.792	1.532–5.189	0.001
PLT ≤ 258 (×10^3^ *μ*l)	3.124	1.708–5.809	<0.001
MPV ≥ 10.6 (fl)	0.532	0.091–2.331	0.437
P-LCR ≥ 28.9 (%)	5.458	1.238–31.722	0.037
MONO ≥ 0.38 (×10^3^ *μ*l)	3.504	1.926–6.478	<0.001
TPO ≥ 42.071 (pg/ml)	4.673	2.620–8.525	<0.001

Significance level: *P* < 0.05; CI: confidence interval; for other abbreviations, see Table 1.

**Table 3:** Correlations between HCV viral load and values for RBC, MCHC, PLT, MPV, P-LCR, MONO, and TPO.

Variables	*r*	*P*
RBC (×10^6^ *μ*l)	0.279	<0.001
MCHC (g/dl)	0.217	<0.001
PLT (×10^3^ *μ*l)	−0.333	<0.001
P-LCR (%)	0.194	0.001
MONO (×10^3^ *μ*l)	0.370	<0.001
TPO (pg/ml)	0.351	<0.001

Significance level: *P* < 0.05; for abbreviations, see Table 1.

**Table 4:** Prediction performance of haematological indices and TPO.

Variables	Cut-off point	AUC	Specificity	Sensitivity	*P*	Score
RBC (×10^6^ *μ*l)	4.76	0.664	0.738	0.521	<0.001	1
MCHC (g/dl)	33.9	0.651	0.601	0.632	<0.001	1
PLT (×10^3^ *μ*l)	258	0.730	0.583	0.771	<0.001	1
P-LCR (%)	28.9	0.655	0.577	0.722	<0.001	1
MONO (×10^3^ *μ*l)	0.38	0.736	0.720	0.667	<0.001	1
TPO (pg/ml)	42.07	0.741	0.643	0.757	<0.001	2

AUC: area under the curve value; for other abbreviations, see Table 1.

## Abbreviations

ALT: Alanine transaminase
ANCOVA: Analysis of covariance
CBC: Complete blood count
ELISA: Enzyme-linked immunosorbent assay
Hb: Hemoglobin
HCC: Hepatocellular carcinoma
HCT: Hematocrit
HBV: Hepatitis B virus
HCV: Hepatitis C virus
MCHC: Mean corpuscular haemoglobin concentration
MONO: Monocyte count
NAT: Nucleic acid test
MPV: Mean platelet volume
PCR: Polymerase chain reaction
P-LCR: Platelet-large cell ratio
PCT: Plateletcrit
PDW: Platelet distribution width
PLT: Platelet count
RBC: Red blood cell count
RIBA: Recombinant immunoblot assay
ROC: Receiver operating characteristic
AUROC: Area under ROC curve
TPO: Thrombopoietin
WBC: White blood cell count.
